# Supplementary material for: Genetic comparisons of interleukin-17 reveal a framework for complex signaling evolution
Source: bioRxiv. 2026 Apr 14:2026.04.13.718218. Preprint. [Version 1] doi: 10.64898/2026.04.13.718218 (PMC13105070; doi:10.64898/2026.04.13.718218)
Supplement: Supplement 4 [file NIHPP2026.04.13.718218v1-supplement-4.pdf]

bioRxiv preprint doi: https://doi.org/10.64898/2026.04.13.718218; this version posted April 14, 2026. The copyright holder for this preprint (which was not certified by peer review) is the author/funder, who has granted bioRxiv a license to display the preprint in perpetuity. It is made available under aCC-BY 4.0 International license.

A

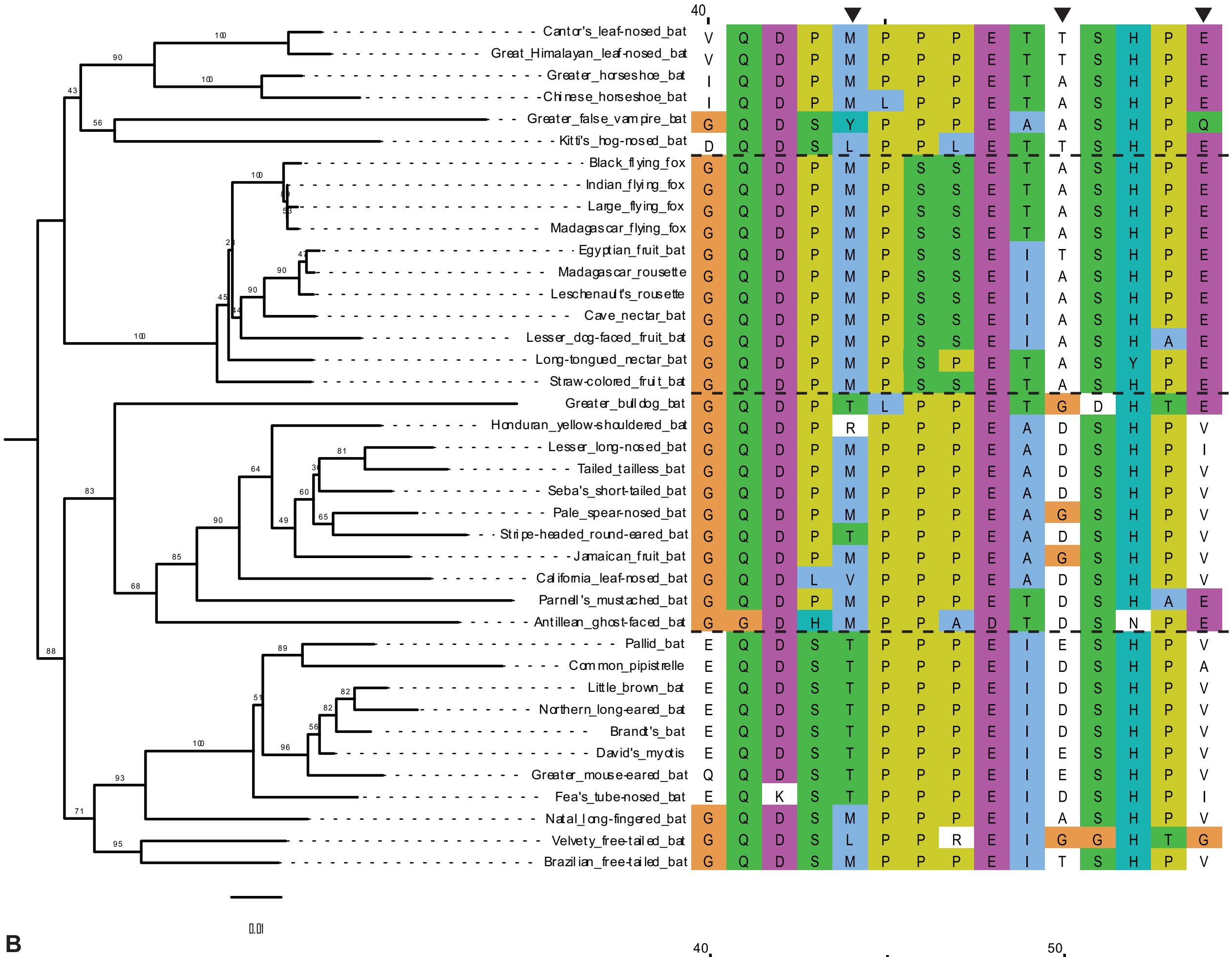

B

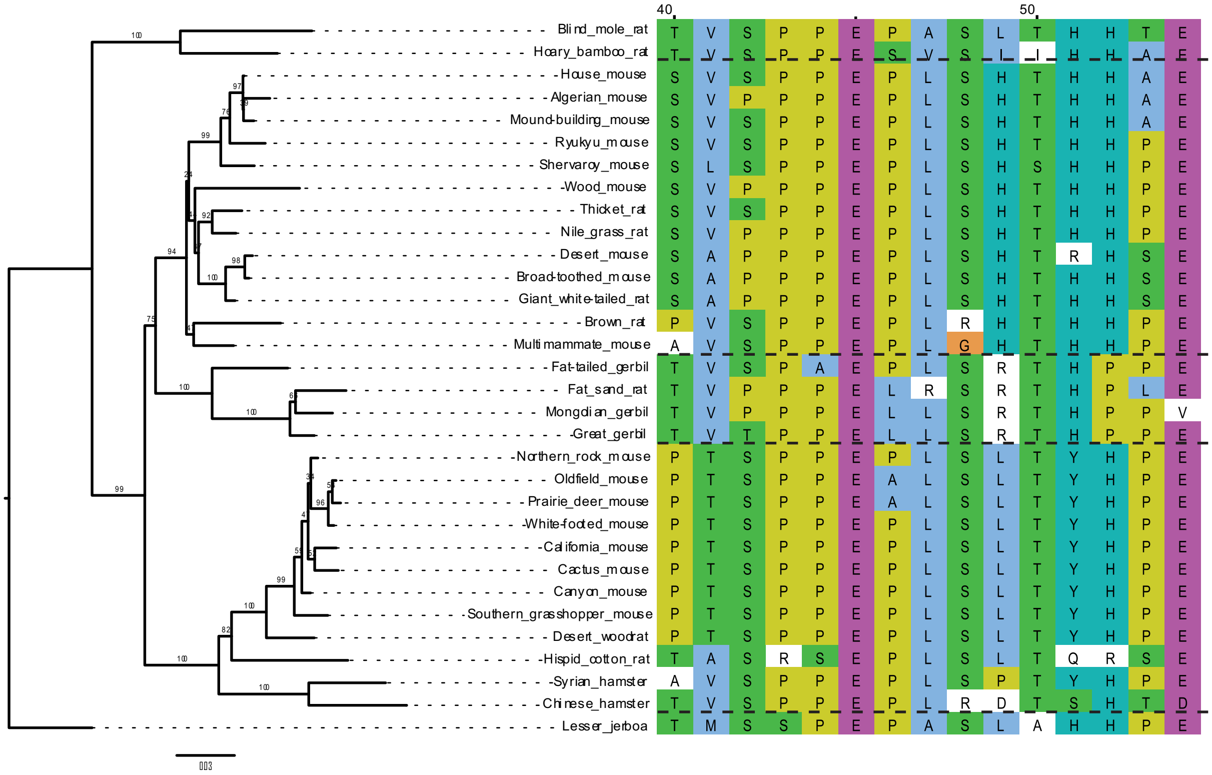

bioRxiv preprint doi: https://doi.org/10.64898/2026.04.13.718218; this version posted April 14, 2026. The copyright holder for this preprint (which was not certified by peer review) is the author/funder, who has granted bioRxiv a license to display the preprint in perpetuity. It is made available under aCC-BY 4.0 International license.

Supplemental Figure 1. Maximum likelihood gene tree of A) bat and B) rodent IL-17E with alignment of 15 amino acid N-terminal disordered region under pervasive positive selection in primates. Black arrows represent residues under rapid evolution as determined by PAML M7-M8 model. PAML did not register rodent IL-17E as evolving under selection.

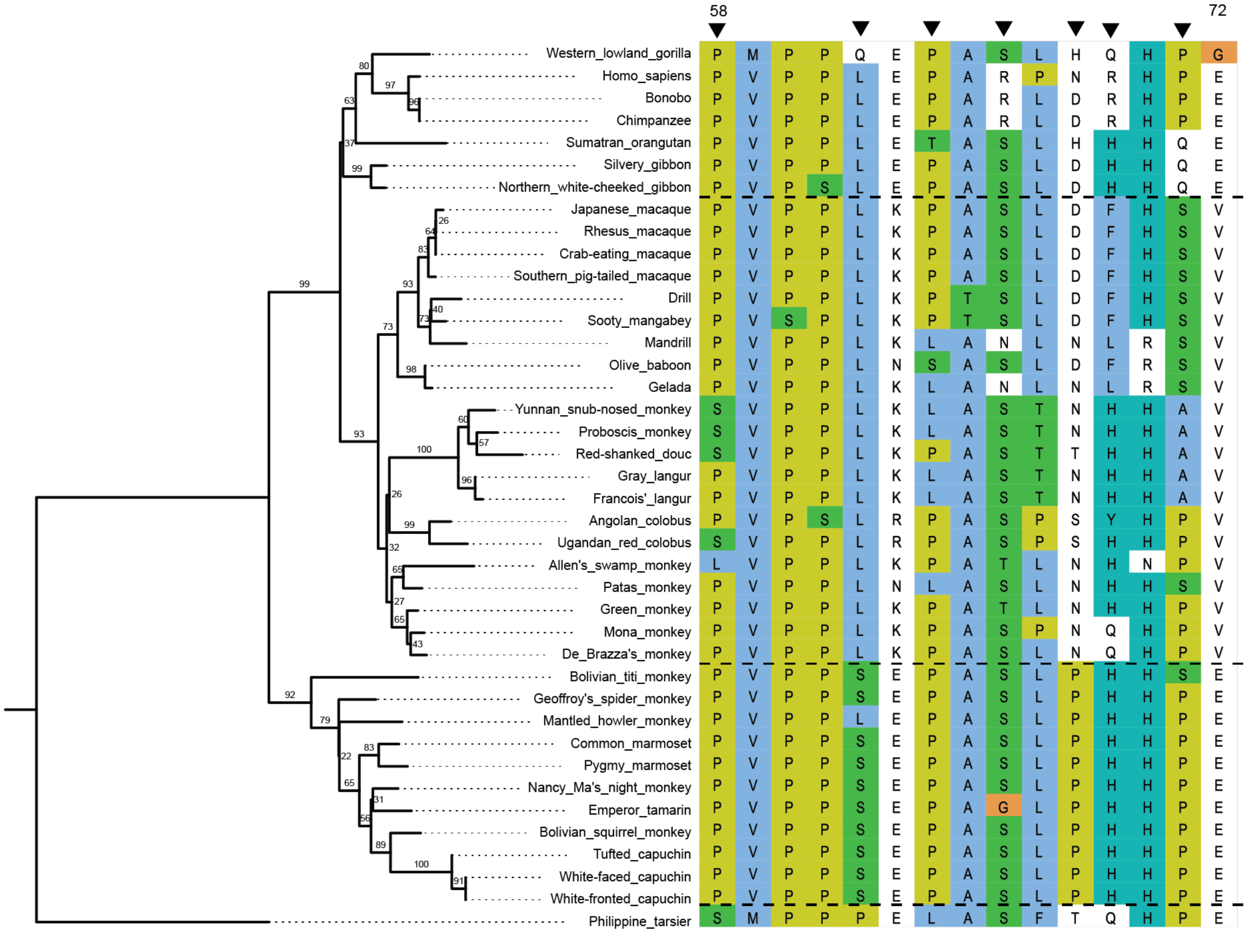

Supplemental Figure 2. Uncollapsed maximum likelihood gene tree of primate IL-17E orthologs used in this study. Alignment window shows the N-terminus disordered domain and black arrows indicate rapidly evolving residues as determined by PAML (BEB > 0.99), MEME (p < 0.05), and/or FUBAR (posterior probability > 0.95).
